# Hierarchical Control on Polyene Macrolide Biosynthesis: PimR Modulates Pimaricin Production via the PAS-LuxR Transcriptional Activator PimM

**DOI:** 10.1371/journal.pone.0038536

**Published:** 2012-06-05

**Authors:** Javier Santos-Aberturas, Cláudia M. Vicente, Tamara D. Payero, Lara Martín-Sánchez, Carmen Cañibano, Juan F. Martín, Jesús F. Aparicio

**Affiliations:** 1 Area of Microbiology, Faculty of Biology, University of León, León, Spain; 2 Institute of Biotechnology INBIOTEC, Parque Científico de León, León, Spain; Belgian Nuclear Research Centre SCK/CEN, Belgium

## Abstract

Control of polyene macrolide production in *Streptomyces natalensis* is mediated by the transcriptional activator PimR. This regulator combines an N-terminal domain corresponding to the *Streptomyces* antibiotic regulatory protein (SARP) family of transcriptional activators with a C-terminal half homologous to guanylate cyclases and large ATP-binding regulators of the LuxR family. The PimR SARP domain (PimR^SARP^) was expressed in *Escherichia coli* as a glutathione S-transferase (GST)–fused protein. Electrophoretic mobility shift assays showed that GST-PimR^SARP^ binds a single target, the intergenic region between the regulatory genes *pimR* and *pimM*s in the pimaricin cluster. The PimR^SARP^-binding site was investigated by DNaseI protection studies, revealing that it contains three heptameric direct repeats adjusting to the consensus 5′-CGGCAAG-3′. Transcription start points of *pimM* and *pimR* promoters were identified by 5′-RACE, revealing that unlike other SARPs, PimR^SARP^ does not interact with the -35 region of its target promoter. Quantitative transcriptional analysis of these regulatory genes on mutants on each of them has allowed the identification of the *pimM* promoter as the transcriptional target for PimR. Furthermore, the constitutive expression of *pimM* restored pimaricin production in a pimaricin-deficient strain carrying a deletion mutant of *pimR*. These results reveal that PimR exerts its positive effect on pimaricin production by controlling *pimM* expression level, a regulator whose gene product activates transcription from eight different promoters of pimaricin structural genes directly.

## Introduction

Streptomycetes are well-known for their ability to produce a great variety of secondary metabolites including therapeutic molecules like polyene macrolide antibiotics. These constitute a large group of antifungal agents [1], [2] whose production, occurs in a growth-phase-dependent manner, at the transition between the rapid growth phase and the stationary growth phase [3]. The control of secondary metabolite production is a rather complex process involving multiple levels of intertwined regulation. Typically the lowest level is composed by pathway-specific transcriptional regulators, which are encoded within the respective biosynthetic gene cluster.

PimR was the first pathway-specific transcriptional regulator of pimaricin biosynthesis to be described [4]. Pimaricin, an archetypical representative of small glycosylated polyenes, is a tetraene produced by *Streptomyces natalensis* [5] whose biosynthetic gene cluster [6]–[11], and other factors regulating production [12], [13] have been characterized. PimR is a transcriptional activator (its inactivation from the *S. natalensis* chromosome resulted in complete loss of pimaricin production [4]) with a peculiar architecture. It contains an N-terminal SARP (Streptomyces Antibiotic Regulatory Protein) domain [14] with a C-terminal half homologous to guanylate cyclases and LAL regulators (Large ATP-binding regulators of the LuxR family) [15]. The C-terminal half includes the ATP/GTP binding AAA domain characteristic of these protein families but lacks the signature sequence at the N-terminus of guanylate cyclases or the LuxR-type helix-turn-helix motif for DNA binding present at the C-terminus of LAL regulators. PimR was the first of its class to be described, and constitutes the prototype of a new class of regulators. Members of this class include the regulator PteR from *S. avermitilis* located in the biosynthetic gene cluster for the pentaene filipin [16], the nikkomycin activator in *S. ansochromogenes* SanG [17], or the polyoxin regulator in *S. cacaoi* PolR [18] which is directly controlled by PolY [19].

SARPs belong to the OmpR family of transcriptional regulators [20]. These proteins have their DNA binding domain at the N-terminus but act as transcriptional activators, unlike most other regulators with such a layout acting as transcriptional repressors [21]. LAL regulators constitute a poorly studied family of transcriptional modulators. Several regulators of this class have been identified in antibiotic and other secondary metabolite gene clusters from actinomycetes [22], [23], thus they have been considered pathway-specific regulators, but it is conceivable that LAL regulators could play a role in higher steps of the regulatory cascade [24].

PimM constitutes the second pathway-specific transcriptional regulator of pimaricin biosynthesis [25]. It also has a peculiar architecture, combining an N-terminal PAS sensory domain [26] with a C-terminal helix-turn-helix motif of the LuxR type for DNA binding. PAS domains were first found in eukaryotes, and were named after their homology to the *Drosophila* period protein (Per), the aryl hydrocarbon receptor nuclear translocator protein (ARNT) and the *Drosophila* single minded protein (Sim). Recently, we characterized the mode of action of PimM at the molecular level, and determined that it binds eight promoters of pimaricin genes [27]. The PimM regulatory model is especially attractive because PimM orthologous regulatory proteins are encoded in all known biosynthetic gene clusters of antifungal polyketides, and all these regulators are functionally conserved [28].

Previous gene expression analyses by reverse transcriptase-polymerase chain reaction (RT-PCR) of the pimaricin gene cluster in a strain carrying a frameshift mutation of the *pimR* gene suggested the targets for the PimR regulatory protein [4]. According to these analyses very low level transcription of key enzyme-encoding genes for pimaricinolide construction except for the mutant *pimR* gene was observed. This result explained the lack of pimaricin production in the mutant, and demonstrated that this regulator activates the transcription of the majority of genes belonging to the pimaricin gene cluster but not its own transcription [4]. Similarly, gene expression analyses by RT-PCR in a strain carrying a deletion of the *pimM* gene revealed its targets, and suggested, erroneously, that both regulators were acting on independent regulatory circuits [25]. Now, electrophoretic mobility shift assays (EMSA), footprinting analyses, quantitative RT-PCR and gene promoter replacement experiments have been used for determining the binding site for PimR and its transcriptional target, thereby elucidating the hierarchical relationship between PimR and PimM.

## Results

### Complete deletion of *pimR* from the *S. natalensis* chromosome blocked pimaricin biosynthesis, and gene complementation restored antifungal production

We had previously constructed a *S. natalensis* Δ*pimR* mutant strain lacking the internal ATP/GTP-binding site of this regulator, but retaining the N-terminal SARP binding domain [4]. In order to avoid interference of this DNA-binding domain with future *in vivo* studies, we decided to construct a new Δ*pimR* mutant where the gene was completely deleted. For that purpose we used the REDIRECT gene replacement technology as indicated in Materials and Methods. Double-crossover mutants were screened by spectinomycin resistance and kanamycin sensitivity. These (about 1%) were verified by both PCR and Southern blot analysis (not shown).

The new strain *S. natalensis* Δ*pimR2* had growth and morphological characteristics identical to those of *S. natalensis* wild type when grown on solid or liquid media, suggesting that PimR has no role in bacterial growth or differentiation. The spore counts of both strains were similar after growth for 9 days at 30°C on TBO plates. The spores of both strains were serially diluted and plated on minimal medium to check their viability, finding no differences between the two strains. Both strains grew well in liquid minimal medium, showing an identical growth curve.

The fermentation broth produced by the new mutant strain, *S. natalensis* Δ*pimR2*, was extracted with methanol and analyzed for the presence of pimaricin. High performance liquid chromatography (HPLC) assays indicated that no pimaricin was being produced by the mutant strain Δ*pimR2* (Fig. S1).

To confirm that the deletion of *pimR* was directly responsible for the abolition of pimaricin production, we complemented the mutant with *pimR*. A DNA fragment containing *pimR* plus its putative promoter region was inserted into the integrative vector pSET152, giving rise to pSETpimR (see Materials and Methods). The plasmid was then transferred from *E. coli* ET12567 [pUZ8002] to *S. natalensis* Δ*pimR2* by conjugation. pSET152 was also introduced into *S. natalensis* wild type as control. Introduction of pSETpimR restored pimaricin biosynthesis to the control levels (Fig. S1). These results were fully consistent with those obtained upon deletion of the *pimR* gene, and confirm the involvement of PimR in pimaricin biosynthesis.

### Heterologous expression of the DNA-binding SARP domain

Heterologous expression of PimR in *E. coli* was first attempted as both N-terminal, and C-terminal 6xHis fusion proteins to facilitate *in vitro* analysis of its function. The coding sequence of *pimR* was cloned into the expression vectors pQE30 and pQE70 (Qiagen), and transformed into *E. coli* BL21(DE3) for expression. Both systems yielded insoluble protein after induction with IPTG. PimR was then expressed as a glutathione S-transferase (GST)-fusion protein following cloning into the pGEX-2T expression vector and transformation into *E. coli* BL21(DE3). Again, the PimR protein obtained was largely insoluble.

These unsuccesful results, which we think could be due to the unusually large size (about 130 kDa) of the protein and the presence in its structure of several putative transmembrane domains, prompted us to express just the SARP DNA-binding domain. Hence, this DNA binding domain was expressed as a 56 kDa glutathione S-transferase (GST)-fusion protein following cloning into the pGEX-2T vector and transformation into *E. coli* BL21(DE3). A significant proportion of GST–PimR^SARP^ fusion protein was found in the soluble fraction, and was purified by glutathione affinity chromatography (Fig. S2). The identity of the fusion protein was verified by MALDI-TOF MS. Purification yielded 4.4 mg of pure protein per liter of *E. coli* culture. This protein was further concentrated by filtration in Amicon tubes.

PimR^SARP^ could not be separated from GST by using thrombin since, regardless of lacking canonical proteolytic sites in its sequence, it got completely degraded upon digestion. However, given that GST-tagged proteins have been successfully used in EMSAs [27], the fusion protein GST- PimR^SARP^ was used for in vitro experiments.

### PimR^SARP^ binds a single target in the pimaricin gene cluster

**Figure 1:**
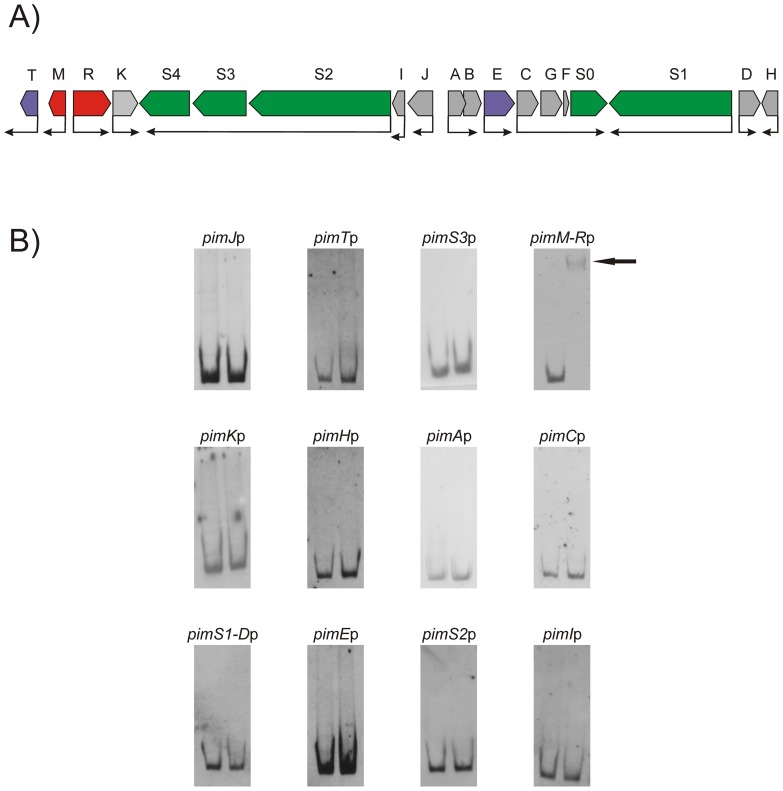
Organization of the pimaricin gene cluster and GST-PimR^SARP^ DNA binding assay results. A) Pointed boxes indicate the direction of transcription. Transcriptional regulatory genes (*pimR* and *pimM*) are indicated in red, other regulators are indicated in purple, and the polyketide synthase genes in green. The remaining genes (in grey) are involved in polyene tailoring or export. B) Electrophoretic mobility analysis (EMSA) of GST-PimR^SARP^ binding to different putative promoter regions. The arrow indicates the DNA–protein complex. Promoter names are indicated above the picture. All experiments were carried out with 2 ng labeled DNA probe. Left lane, control without protein; right lane, 10 µM of GST-PimR^SARP^ protein.

**Figure 2:**
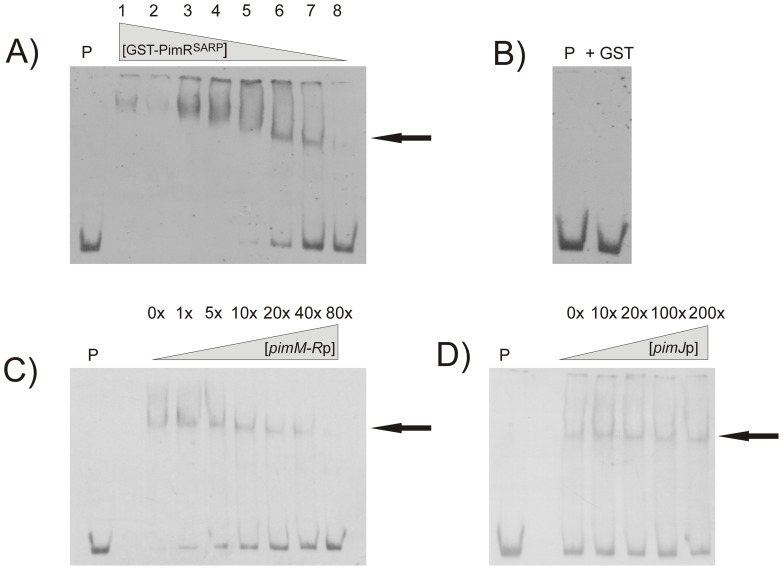
Binding of GST-PimR^SARP^ to its target is specific. Analysis by EMSA of the binding of GST-PimR^SARP^ to the *pimR-M* promoter region. Arrows indicate the DNA–protein complexes. All experiments were carried out with 2 ng labeled DNA probe. Lane P, control without protein. A) Decreasing gradient of protein. Lane 1, 5 µM of protein; lane 2, 2.5 µM; lane 3, 1.25 µM; lane 4, 625 nM; lane 5, 312 nM; lane 6, 160 nM; lane 7, 80 nM; lane 8, 40 nM of protein. B) Control reaction with 10 µM of pure GST protein. C) Competition experiment between labeled *pimM-R* promoter and unlabeled *pimM-R* promoter. The experiment was performed with 1.25 µM of GST-PimR^SARP^. D) Competition experiment between *pimM-R*p and *pimJ*p. Note that 200-fold-higher concentrations of unlabeled *pimJ*p competitor DNA failed to decrease the intensities of the *pimM-R*p retardation bands. The experiment was performed with 80 nM of GST-PimR^SARP^.

As shown in Figure 1, 12 different DNA probes containing all the known promoter regions of the pimaricin gene cluster (Fig. 1A) were tested in the search for direct interactions with the SARP domain of PimR by EMSA. No interaction was observed for any of the probes that contained the promoter regions of the biosynthetic pimaricin genes, the non-transcriptional regulatory genes, or the transporters (Fig. 1B). However, a strong band shifting was observed with the probe containing the *pimM*-*pimR* intergenic region (Fig. 1B). In this case, a progressive decrease in the amount of added GST-PimR^SARP^ protein resulted in the progressive decrease of the intensity of the retarded band (Fig. 2A). To discard the possibility that such interaction could be produced by the GST moiety of the fusion protein, an EMSA assay was performed in the same conditions but using pure GST (60 μM) instead of GST-PimR^SARP^. This experiment was negative, excluding a possible binding of the GST protein to the promoter, (Fig. 2B).

To ensure that the binding of GST-PimR^SARP^ to *pimM*-*R*p was specific, competition experiments in which different unlabeled probes were added to the usual binding reaction were performed. As shown in Figure 2C, the addition of an increasing amount of *pimM*-*R*p unlabeled probe resulted in a progressive decrease of the retarded band intensity. In contrast, the addition of increasing amounts of an unlabeled promoter region such as *pimJ*p, failed to diminish the intensity of the retardation band (Fig. 2D).

Taken together, these results indicate that GST-PimR^SARP^ interacts directly with the intergenic region between *pimR* and *pimM*, and does it in a specific way.

### DNaseI protection studies reveal PimR^SARP^ binding site

To determine the PimR^SARP^ binding sequence, the promoter region shown above to be retarded in EMSA was studied by DNase I protection analysis. GST–PimR^SARP^ protein (10 µM) was tested using a 5′-end fluorescein-labeled DNA fragment. All analyses were carried out by triplicate.

**Figure 3:**
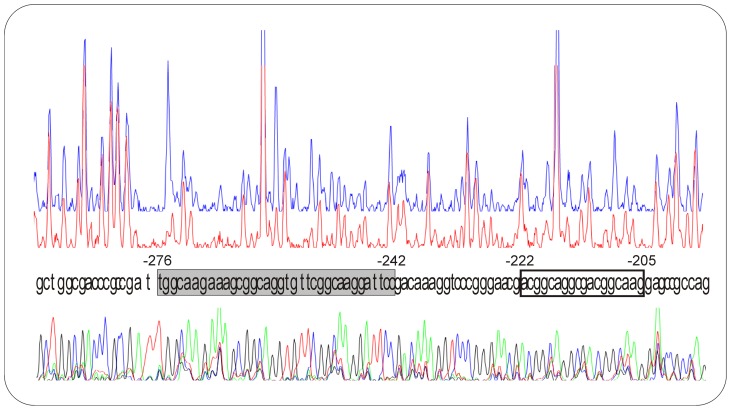
Identification of binding sites. The upper electropherogram (blue line) is the control reaction. The main protected nucleotide sequence is boxed in grey, a secondary group of protected nucleotides is boxed in white. Sequencing reactions are included. Coordinates are from the translation start point.

Results showed a major protected region extending for 35 bp of *pimM* coding strand (Fig. 3), in agreement with the appearance of one retardation band in EMSA experiments. This protected region is located at nucleotide positions −276 to −242 with respect to the *pimM* translational ATG start site (positions −155 to −122 from the *pimR* translational start site). Interestingly, the nucleotide sequence of this protected region (TGGCAAGaaagCGGCAGGtgttCGGCAAGgattcc) contains three heptameric direct repeats (in uppercase) with 4 bp spacers. Heptameric repeats are typical for SARP-binding targets, and in this case are almost coincident with those recognized by the nikkomycin regulator SanG [29], and the polyoxin activator PolR [18] which is directly controlled by PolY [19]. Strikingly, while SanG and PolR bind a region with two direct repeats, PimR^SARP^ binding region contains three of such repeats.

Some protected nucleotides were also observed downstream the main protected stretch (Fig. 3). This region also contains two heptameric direct repeats (aCGGCAGGcgaCGGCAAG), although in this case the spacing is of 3 nucleotides. This feature might explain the weak protection observed in this region, and also the absence of an additional retardation band in the EMSA assays.

### Characterization of *pimR* and *pimM* promoters

**Figure 4:**
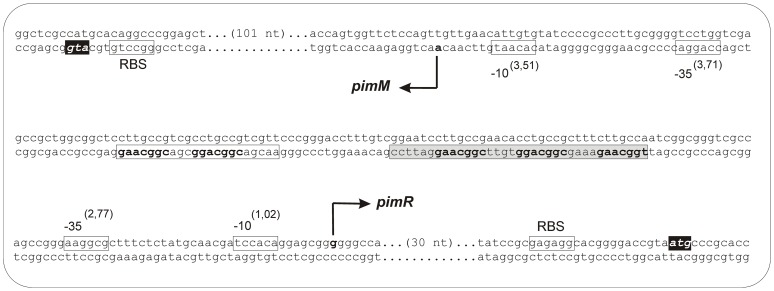
Transcriptional start sites of *pimR* and *pimM*. The position of the transcriptional start site was determined by 5′ RACE. The putative −10 and −35 hexanucleotides are boxed. Scores resulting from the comparison to the matrices reported by Bourn and Babb [30] for *Streptomyces* are indicated between brackets. The TSP is indicated by a bent arrow and bold type letter. Nucleotides showing homology with the 16S RNA, which could form a ribosome-binding site, are framed with a box labeled RBS. The start codon is shown in a black box. The main protected nucleotide sequence is indicated with a shaded box, and the secondary group of protected nucleotides is boxed in white. The heptameric repeats are indicated in bold.

To determine the transcriptional start sites of *pimM* and *pimR* promoters, 5′-RACE experiments were carried out. Once the +1 sites were known, the corresponding −10 and −35 boxes of each promoter were established by comparing them to the matrices reported by Bourn and Babb [30] for *Streptomyces* that take into account the nucleotides occurring in 13-nucleotide stretches, including the –10 or –35 consensus hexamers (see Materials and Methods). Results are summarized in Fig. 4.

A single RACE product of approximately 400 bp was observed for *pimM*. The *pimM* transcription start point (TSP) is located at an adenine at 148 bp upstream from the ATG codon. Analysis of the region upstream of the TSP revealed that the −10 box with the highest score to the consensus *Streptomyces* was CACAAT (score 3.51), centered at 10 nucleotides from the start site. A search using combined class C–class A matrices [30] revealed a −35 box CCAGGA separated by 19 nucleotides, with a score of 3.71. Noteworthy, the protected region observed in the footprinting assays is 93 nt away from the TSP site, and does not cover the −35 hexamer box (Fig. 4).

For *pimR*, a single RACE product of *ca*. 350 bp was observed. Its TSP corresponds to a guanine located 63 bp upstream from the ATG codon (Fig. 4). The sequence TCCACA (score 1.02) centered at position −10, constitutes the −10 consensus, and a −35 box AAGGCG (score 2.77) was identified at 17 nt distance. As in the former case, the protected region lays 58 nt upstream from the TSP site, and does not cover the −35 hexamer box of the promoter (Fig. 4). This is unsual since DNA-binding domains of SARP regulators always bind sequences that overlap the −35 hexamer of the promoters they control [18], [29], [31]–[33].

### PimR is the hierarchical superior that controls *pimM* transcription

Above results indicated that PimR interacts directly with the *pimM*-*pimR* intergenic region, binding an operator containing heptameric direct repeats, which is typical of SARP-binding targets [33], but unlike other SARPs this sequence did not overlap the −35 element of any of the two promoters present in that region. In order to determine the target promoter of PimR, we studied the expression of both *pimR* and *pimM* genes in *S. natalensis* Δ*pimR2*, and also in a Δ*pimM* mutant [25], and compared them with the parental strain.

**Figure 5:**
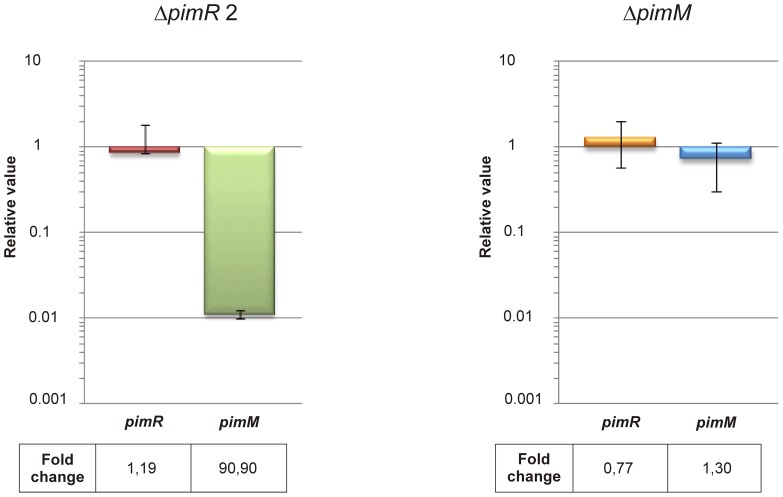
PimR controls *pimM* transcription. Gene expression was assessed using quantitative RT-PCR with the primers indicated in Table S2. The relative values are referred to 1, the assigned relative value for the expression of each gene in *S. natalensis* ATCC 27448. The expression of *rrnA1* (encoding 16S rRNA) was used as control. Error bars were calculated by measuring the standard deviation among two biological and six technical replicates of each sample. The mRNA templates were from 48 h cultures grown in YEME medium without sucrose. Fold change values are indicated below.

Total RNA was prepared from *S. natalensis* wild type and mutants Δ*pimR2* and Δ*pimM* after growth for 48 h (when pimaricin is actively produced [6]) and used as template for gene expression analysis by quantitative RT-PCR. The expression levels of *pimR* and *pimM* genes in both mutants in relation to those of the wild-type strain (assigned a relative value of 1) are shown in Figure 5. Transcription levels of 0.84 and 1.29 were found for *pimR* in the mutants Δ*pimR2* and Δ*pimM* respectively (Fig. 5) indicating that the expression of this regulator is not affected by any of the mutations in a statistically significant level (see Materials and Methods), and confirming that PimR is not autoregulated. However, when we analysed *pimM* transcription in the same strains, while its expression was not affected (relative value of 0.72) in *S. natalensis* Δ*pimM*, indicating that PimM is not autoregulated, it was dramatically reduced in the Δ*pimR2* mutant, showing a relative value of 0.011 (Fig. 5). This means 90-fold less expression than in the parental strain, and clearly indicates that *pimM* promoter is the transcriptional target of PimR.

**Figure 6:**
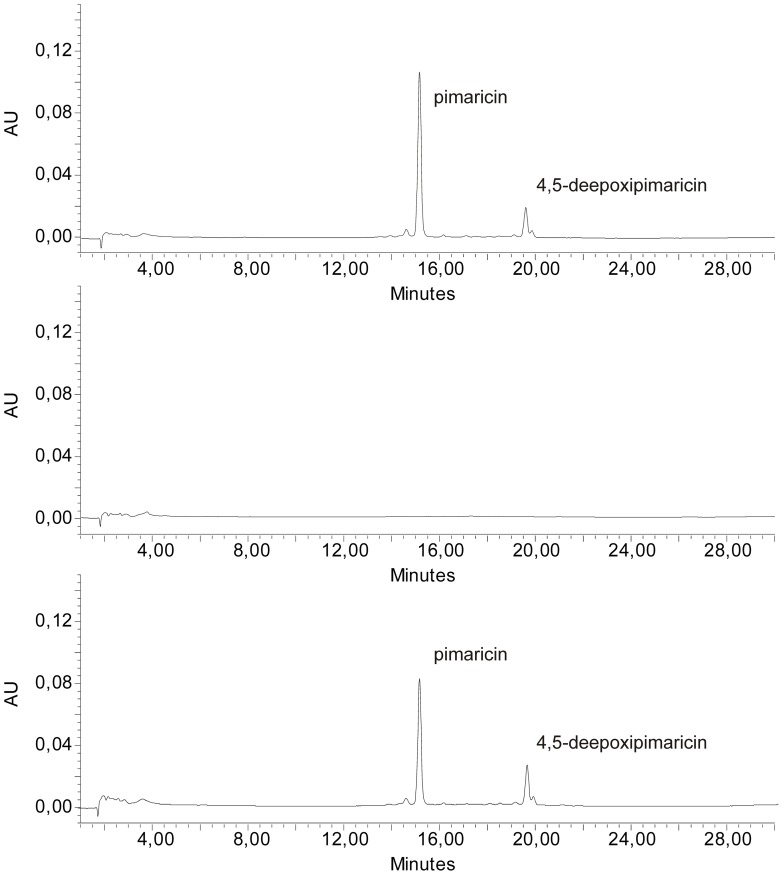
Constitutive expression of *pimM* in *S. natalensis* Δ***pimR2*** **results in restoration of pimaricin production.** Comparison of HPLC analyses of methanol-extracted broths from *S. natalensis* wild type (top), Δ*pimR2* (middle), and Δ*pimR2* + pCPpimM strains (bottom). Detection was carried out at A_304nm_. Chromatographic peaks corresponding to pimaricin and 4,5-deepoxypimaricin are indicated by arrows.

In order to corroborate this finding, we introduced *pimM*, under the control of a constitutive promoter, into *S. natalensis* Δ*pimR2.* We used the ermE* promoter, an upregulated variant of *ermE* promoter that has been frequently deployed as a strong constitutive promoter for gene expression in *Streptomyces*. For this purpose, a DNA fragment containing *pimM* plus its ribosomal binding site was inserted into the integrative vector pIB139 [34], giving rise to pCPpimM (see Materials and Methods). This construct, in which *pimM* is placed under the control of the ermE* promoter but uses its own ribosome binding site, was then transferred from *E. coli* ET12567 [pUZ8002] to *S. natalensis* Δ*pimR2*. The constitutive expression of *pimM* bypassed the *pimR* mutation and restored pimaricin biosynthesis (Fig. 6), thus demonstrating that the *pimM* promoter is the unique target for PimR.

## Discussion

The regulator PimR contains an N-terminal domain corresponding to the SARP family of transcriptional activators with a C-terminal half homologous to guanylate cyclases and large ATP-binding regulators of the LuxR family. Regulators with a similar architecture include the putative biosynthetic regulator PteR involved in filipin biosynthesis in *S. avermitilis* [16], the nikkomycin activator SanG in *S. ansochromogenes* [17], and the polyoxin regulator in *S. cacaoi* PolR [18]. Filipin is a polyene macrolide while nikkomycins and polyoxins are peptidyl nucleoside antibiotics. Interestingly, although these compounds are structurally different, and have a different mode of action [1], all of them are effective antifungals. It seems plausible that these regulators with highly similar architectures could share similar regulatory mechanisms. Since their only common feature is their antifungal activity, it is tempting to speculate that their domain arrangement might be related with the detection of common signals involved in the triggering of antifungal production.

Electrophoretic mobility shift assays have been used here to prove the direct binding of the PimR SARP domain to the intergenic region between *pimM* and *pimR*, and that this is the unique target of the regulator within the pimaricin biosynthetic gene cluster. Quantitative RT-PCR was then used to show the dependence of *pimM* expression on the presence of intact PimR, thus placing the latter protein above PimM in regulatory hierarchy. This result was confirmed by replacing *pimM* promoter by a constitutive promoter, such as ermE*, in the pimaricin-deficient strain *S. natalensis* Δ*pimR2*, and restoration of pimaricin production. Taken together, these results demonstrate that the positive effect exerted by PimR on pimaricin production takes place via the regulation of *pimM* expression level.

Analysis of the protected sequence in the DNase I protection assays revealed PimR^SARP^ binding site, showing that it contains three heptameric direct repeats of the consensus CGGCAAG with 4 bp spacers. Operators with heptameric repeats separated by four-nucleotide spacers positioned on the same face of the DNA helix (one complete turn of the DNA helix) are typical of SARP regulators [14], although the precise consensus nucleotide sequence of the heptamer, and the number of repetitions, varies depending on the regulator [33]. Strikingly, the consensus heptamer for PimR^SARP^ is identical to those of SanG [29] and PolR [18], although in these cases only two heptameric repeats are present in the operator. It could be argued that the lack of the rest of the protein might be affecting specificity and degree of binding. In our opinion, this is unlikely, since in other multidomain SARP regulators such as AfsR, it has been demonstrated that the specificity of binding relies exclusively in the SARP domain [33]. However, at this stage such possibility cannot be excluded.

Analysis of PimR^SARP^ binding site together with the identification of the transcriptional start points of *pimM* and *pimR* promoters revealed that unlike all SARPs, including PimR counterparts SanG and PolR, PimR does not interact with the −35 region of its target promoter. Instead, its binding site is located at 55 nucleotides upstream of that element. To our knowledge, this is unprecedented for a SARP regulator, since these regulators always interact with the −35 element of target promoters [33]. This result must be taken with some reservation, given that our assays have been carried out with just the DNA-binding domain of PimR. However, no heptameric repeats are present overlapping the −35 hexamer of *pimM* promoter.

PimR-DNA interaction is presumed to enable protein-protein contacts between RNA polymerase and PimR as an important functional aspect in transcriptional activation. This would correspond to a Class I activation mechanism where PimR would contact the C-terminal domain of the RNA polymerase α subunit, resulting in recruitment of the RNA polymerase holoenzyme to the promoter [35]. Future experimental analyses will be required to test this hypothesis.

The possible role of the secondary protected region observed in the footprinting assays is intriguing. This region contains two consensus heptameric repeats, but the spacer is of only 3 nucleotides, thus conforming 10 bp repeating units instead of the classical 11 bp SARP binding sequences [14]. In any case, this weak-protected sequence is still far away from the −35 element of the *pimM* promoter (18 pb) and does not overlap it, so its presence and hypothetical functionality (that will be the focus of future experimental efforts) would not essentially alter the proposed transcriptional activation model.

Notably, unlike its counterparts SanG and PolR, which exert their regulatory effects by directly interacting with promoters of structural genes [18], [29], PimR acts as a regulator of regulators, modulating the expression level of *pimM*. PimM, in turn, controls the expression of the genes *pimK*, *pimS2S3S4*, *pimI*, *pimJ*, *pimAB*, *pimE*, *pimS1*, and *pimD* through direct binding to the promoters of these genes [27]. Taken together, these results reflect a very clear regulatory cascade, in which PimR regulates the transcription of *pimM*, which in turn activates the transcription of pimaricin biosynthetic genes from eight different promoters [27]. In this model, PimR and PimM represent two different but consecutive points of control for pimaricin production. Although some cases of SARPs acting as regulators of regulators are known [36], PimR represents the first example belonging to the SARP-LAL subfamily of regulatory proteins. The different type of targets between PimR and its counterparts SanG and PolR illustrates the flexibility in which evolution is able to arrange the components of regulatory cascades in order to achieve the best adaptive responses to environmental challenges.

Unlike PimM, which binds multiple promoters and whose expression constitutes a bottleneck for pimaricin production [28], PimR has a single target, so it is unlikely that its expression could constitute a bottleneck for biosynthesis. In fact, the introduction of an extra copy of *pimR* into *S. natalensis* has no effect on pimaricin production [37]. In contrast, *sanG* [17] or *polR* [18] gene copy increments boost the production of nikkomycins and polyoxins, respectively.

Interestingly, the binding sequence of PimR (TGGCAAGaaagCGGCAGGtgttCGGCAAG) is exactly conserved in the intergenic region between between *scnRII* and *scnRI* in the pimaricin gene cluster of *S. chattanoogensis* (*pimM* and *pimR* counterparts, respectively [38]), and also between *pteF* and *pteR*, the corresponding counterparts in the filipin gene cluster of *S. avermitilis*, including the inter-heptamer nucleotides. These genes are also arranged divergently in the chromosome. It is thus likely that the hierarchical relationship between PimR and PimM could be conserved in other polyene regulatory pathways.

## Materials and Methods

### Bacterial strains and cultivation

*S. natalensis* ATCC 27448 was routinely grown in YEME medium without sucrose [39]. Sporulation was achieved in TBO medium [39] at 30°C. *Escherichia coli* strain DH5α was used as a host for DNA manipulation. *E. coli* BL21 (DE3) was used for expression studies. *E. coli* ET12567 [pUZ8002] was used as donor in intergeneric conjugations.

### Plasmids and DNA manipulation procedures

pUC19 (New England Biolabs) was used as the routine cloning vector, pGEX-2T (GE-Healthcare) was the vector used to construct expression plasmids, and pSET152 (Am^R^, pUC18 replicon, ΦC31 *attP* [40]) and pIB139 [34] the vectors used for gene complementation. Plasmid and genomic DNA preparation, DNA digestion, fragment isolation, and transformation of *E. coli* were performed by standard procedures [41]. Polymerase chain reactions were carried out using Phusion DNA polymerase as described by the enzyme supplier (Finnzymes). DNA sequencing was accomplished by the dideoxynucleotide chain-termination method using the Perkin Elmer Amplitaq Gold Big Dye-terminator sequencing system with an Applied Biosystems ABI 3130 DNA genetic analyzer (Foster City, CA., USA). DNA delivery into *Streptomyces* strains was accomplished by intergeneric conjugation as described [42].

### Deletion of *pimR*

Deletion of *pimR* of *S. natalensis* was made by replacing the wild-type gene with a cassette containing a spectinomycin selective marker using a PCR based system [43]. The plasmid pIJ778 containing the spectinomycin resistance gene (*aadA*) and the *oriT* replication origin was used as a template. The mutant was constructed using the oligonucleotides 5′-cagtcagccatatccgcgagaggcacggggaccgtaatgATTCCGGGGATCCGTCGACC-3′ and 5′-*cctccctttgatgtcacggcgggcgtcgggaaatccttat*GTAGGCTGGAGCTGCTTC-3′ as the forward and reverse primers respectively (the sequence identical to the DNA segment upstream from the start codon of *pimR* is underlined and in lower case and the sequence identical to the segment downstream from the stop codon of *pimR* is in lower case italics). These two long PCR primers (59 nt and 58 nt) were designed to produce a deletion of *pimR* just after its start codon leaving only its stop codon behind. The 3′ sequence of each primer matches the right or left end of the disruption cassette (the sequence is shown uppercase in both primers). The extended resistance cassette was amplified by PCR and *E. coli* BW25113/pIJ790 bearing cosmid P6 [39] was electro-transformed with this cassette. The isolated mutant cosmid was introduced into non-methylating *E. coli* ET12567 containing the RP4 derivative pUZ8002. The mutant cosmid was then transferred to *S. natalensis* by intergeneric conjugation [42]. Double cross-over exconjugants were screened for their kanamycin sensitivity and spectinomycin resistance.

### Constructs for gene complementation

In order to complement Δ*pimR2* replacement mutant, pNAF1 [4] was digested with *Sac*I and *Kpn*I to generate a 3874 bp fragment which was cloned into the *Sac*I and *Kpn*I sites of pUC18, resulting in pNAF1B. Separately, pNAF1 was cut with *Not*I and *Sac*I to yield a 541 bp fragment which was cloned into the *Not*I and *Sac*I sites of pUC18, resulting in pNAF1A. Then, a 538 bp *Eco*RI and *Sac*I fragment from pNAF1A was cloned into the same sites of pNAF1B to generate pNAF3. Finally, a 4403 bp *Bam*HI DNA fragment containing the entire *pimR* gene including its own promoter was obtained from pNAF3 and ligated into a *Bam*HI-cut pSET152, to yield pSETpimR. This plasmid was then transferred by conjugation from *E. coli* ET12567 [pUZ8002] to the *S. natalensis* Δ*PimR2* mutant as previously described [42].

### Polyene production assessment

To assay pimaricin in culture broths, 0.5 ml of culture was extracted with 4 ml of methanol, and further diluted with methanol to bring the absorbance at 319 nm in the range of 0.1 to 0.4 units. Control solutions of pure pimaricin (Sigma) were used as control. To confirm the identity of pimaricin, an UV-visible absorption spectrum (absorption peaks at 319, 304, 291 and 281 nm) was routinely determined in a Hitachi U-2900 spectrophotometer. Quantitative determination of pimaricin was performed as previously described [44], using a Mediterranea Sea C18 column (4.6×150 mm, particle size, 3 µm) (Teknokroma).

### Expression and purification of GST fusion protein

PimR SARP domain (PimR^SARP^) was overexpressed in E. coli BL21(DE3) cells as a GST fusion protein. Expression vector was constructed based on the pGEX-2T (GE-Healthcare) vector using PCR. The forward primer used (5′-TACAGGATCCATGCCCGCACCACCGACCGC-3′) introduced a unique BamHI site at the 5′ end of the gene, while the reverse primer (5′-TACGGAATTCTTCTAGGGGGCGCTCGCTCC-3′) carries an EcoRI site. This generates a GST-PimR^SARP^ fusion protein which includes the first 281 residues of PimR (SARP domain). The amplified DNA fragment was digested with BamHI and EcoRI and cloned into the same sites of pGEX-2T to generate pPimR^SARP^. The amplified DNA fragment was sequenced from the expression vector in order to discard any mistakes introduced by the DNA polymerase.

*E. coli* transformants were grown at 18°C in 600 ml LB medium containing 100 μg/ml of ampicillin until an OD_600_ of 0.7 was reached and then induced by adding isopropyl 1-thio-β-D-galactopyranoside to a final concentration of 0.1 mM, and grown for an additional 14 h at 18°C. Cells were harvested, resuspended in PBS buffer pH 7.3, and lysed by sonication using an ultrasonic processor XL apparatus (Misonix Inc.). The insoluble material was separated by centrifugation, and the soluble fraction was filtered and applied to a Glutathione sepharose 4B (Pharmacia biotech) column. Protein was eluted with 10 mM reduced glutathione in 50 mM Tris-HCl pH 8.0, and conserved in 20% glycerol at −80°C before use. Protein elution was monitored at 280 nm and the presence of the fusion protein was assessed by SDS-PAGE.

PimR^SARP^ could not be separated from GST by using thrombin since, regardless of lacking canonical proteolytic sites in its sequence, it got completely degraded upon digestion. However, given that GST-tagged proteins have been successfully used in EMSAs [27], [45], we decided to use the fusion protein GST- PimR^SARP^ for in vitro experiments.

### DNA-protein binding assays

DNA binding tests were performed by EMSA. The DNA fragments used for EMSA were amplified by PCR using the primers as described [27], and labeled at both ends with digoxigenin with DIG Oligonucleotide 3′-End Labeling Kit, 2nd Generation (Roche Applied Science). Binding assays were performed with the GST–PimR^SARP^ protein (40 nM–10 μM) using the same buffer conditions described by Li *et al* [18] for the binding reactions of PolR. The final binding reaction mixture was 10 mM Tris-HCl pH 7.5, 5 mM MgCl_2_, 2 mM dithiothreitol, 7.8 mM glutathione, 40 mg/ml poly dI-dC, 17% glycerol and 0.5 mg/ml BSA in a final volume of 25 μl.

### Footprinting assays

DNase I footprinting assays were performed by the fluorescent labelling procedure as described in Santos-Aberturas *et al*. [27], using the same binding conditions as for the EMSA assays. The DNA fragment used was the same as the one used for EMSA experiments, cloned into pUC19, and amplified by PCR using the universal and reverse primers, one of them labeled with 6-carboxyfluorescein. The same labeled oligonucleotide served to prime the sequencing reaction. The PCR product was purified after agarose-gel electrophoresis and DNA concentrations were determined with a NanoDrop ND-1000 spectrophotometer (Thermo Scientific).

DNase I footprinting was performed by incubating 0.28 pmol of the DNA probe and 10 µM GST–PimR^SARP^ protein for 10 min at 30°C. Lyophilized bovine pancreas DNase I (Roche grade I) was reconstituted in 20 mM Tris HCl pH 7.0, 50 mM NaCl, 100 µg/ml BSA, 1 mM DTT, 10% glycerol to a final concentration of 2.5×10^−3^ units/µl. Nuclease digestions were carried out with 0.01 units (4 µl) at 30°C for 1 min and stopped with 120 µl of 40 mM EDTA in 9 mM Tris HCl pH 8.0. After phenol-chloroform purification and ethanol precipitation, samples were loaded in an Applied Biosystems ABI 3130 DNA genetic analyzer (Foster City, CA., USA). Results were analysed with the PEAK SCANNER program (Applied Biosystems).

### Bioinformatic analysis

Candidate sequences to contain promoters were analyzed using the Patser algorithm [46], implemented in the web resource Regulatory Sequence Analysis Tools [47]. The pseudocount value was set to 10, and the alphabet parameter was adjusted to the GC content of *Streptomyces* genome: AT, 0.15; CG, 0.35. The matrices used to search for regions −35 and −10 were those derived from the alignments of class C and class A promoters of Bourn and Babb [30]. To search for a combination of ‘class C–n nucleotides of separation–class A’, we included n columns of null values in the combined matrix.

### Isolation of total RNA

*S. natalensis* ATCC 27448 was grown for 48 h in YEME medium without sucrose (stationary phase of growth), the cultures were then treated as described elsewhere [48].

### Rapid amplification of cDNA ends (RACE)

The 5′ ends of transcripts were identified by using a 5′ RACE system for rapid amplification of cDNA ends kit (Invitrogen) following the manufacturer's instructions (version 2.0). Briefly, first strand cDNA synthesis was carried out using 3.7 µg of RNA, reverse transcriptase, and the gene specific primer (numbers 1 in supp. Table S1). The cDNA was purified using the SNAP columns provided in the kit, and poly(dC) tails were added to the 3′ ends using terminal deoxynucleotidyl transferase. PCR amplification of the tailed cDNA was carried out using the 5′ RACE abridged anchor primer with the first nested primer (numbers 2 in supp. Table S1). A dilution of the PCR mixture was then subjected to reamplification using the abridged universal amplification primer with the second nested primer (numbers 3 in supp. Table S1). The PCR products were gel-purified and sequenced. When cDNA tailing with poly(dC) did not permit the identification of the transcription start point, poly(dA) tails were added to the 3′ ends of cDNA. In these cases, second strand cDNA synthesis was necessary prior to nested amplifications and was carried out using the 3′ RACE adapter primer (invitrogen). PCR amplification of the cDNA was then carried out using the abridged universal amplification primer with the first nested primer (numbers 2 in supp. Table S1). Final nested amplification was carried out as before.

### Quantitative real-time PCR

Reverse transcription of total RNA was performed on selected samples with 5 µg of total RNA and 12.5 ng/µl of random hexamer primer (Invitrogen) ausing SuperScript™ III reverse transcriptase (Invitrogen) according to manufacturer's instructions. All RNA samples were analyzed with the Agilent 2100 Bioanalyzer (Agilent Technologies, Palo Alto, CA) and only those with RIN values [49] raging from 6.5–7.5 were selected. Each reaction was performed in 20 µl with SYBR® Premix Ex Taq^TM^ (TaKaRa), 200–300 nM of each primer and the template cDNA 1∶2 diluted and run on a StepOnePlus Real Time PCR system (Applied Biosystems). Reactions were carried out on two biological replicates with six technical replicates each and appropriate controls were included to verify the absence of gDNA contamination in RNA and primer-dimer formation. Primers (see supp. Table S2) were designed to generate PCR products between 62 and 137 bp, near the 5′-end of mRNA using the PRIMER3 software [50]. The PCR reactions were initiated by incubating the sample at 95°C for 10 min followed by 40 cycles at 95°C for 15 s, 66–70°C (depending of the set of primers used) for 34 s. To check the specificity of real-time PCR reactions, a DNA melting curve analysis was performed by holding the sample at 60°C for 60 s followed by slow ramping of the temperature to 95°C. SYBR fluorescence was normalized by ROX fluorescence. Baseline and threshold values were determined by the StepOnePlus software. C_t_ values were normalized with respect to 16S rRNA (*rrnA1*). Relative changes in gene expression were quantified using the Pfaffl method [51] and the REST© software [52]. The corresponding real-time PCR efficiency (E) of one cycle in the exponential phase was calculated according to the equation E 10^[−1/slope]^ [53] using 5-fold dilutions of genomic DNA raging from 0,013 to 40 ng (n = 6 with three replicates for each dilution) with a coefficient of determination *R* ^2^>0,98 (Fig. S3).

### Construct for the constitutive expression of *pimM*

In order to corroborate that *pimM* promoter was the only target for PimR, and to establish the hierarchical relationship between both regulators, we introduced *pimM*, under the control of a constitutive promoter, into *S. natalensis* Δ*pimR2*. For that purpose, a 698 bp DNA fragment containing the entire *pimM* gene including 92 bp upstream from the start codon (thereby including its ribosome binding site) was amplified by PCR with primers PMRBSD (5′-TACAGGATCCGCTTGCCAGCCTCCGAATTGAC-3′) and PMRBSR (5′-GGAATTCGCCTGTGCCCGCTCACTTCACG-3′). The PCR product was digested with both *Bam*HI and *Eco*RI and ligated into the same sites of pIB139 (Am^R^, pUC18 replicon, ΦC31 *attP*; [34]), to yield pCPpimM. This plasmid that contains *pimM* under the control of the constutive promoter ermE*, and includes the original ribosome binding site of *pimM*, was then transferred by conjugation from *E. coli* ET12567 [pUZ8002] to the *S. natalensis ΔPimR2* mutant as previously described [42].

## Supporting Information

Figure S1 **Gene complementation of** ***S. natalensis ΔpimR2*** **mutant restores pimaricin biosynthesis.** Quantification of the pimaricin production attained by the complemented strain after 60 and 84 hours of growth. Data are the average of three flasks. Vertical bars indicate the standard deviation values.
(TIF)

Figure S2 **Purification of GST-fusion protein in** ***E.coli*** **BL21.** Purification of GST-PimR^SARP^ by affinity chromatography on Glutathione Sepharose. Lane T, total *E. coli* cell extract; lane P, purified proteins after affinity chromatography. Left lane, molecular size markers (in kDa).
(TIF)

Figure S3 **Primer efficiency.** The efficiency of each set of primers was calculated according to the equation E = 10^[−1/slope]^−1. Using 5-fold dilutions of genomic DNA, the resulting Ct values were plotted against the logarithm of the DNA quantity as shown in A (primers for *pimM*), B (primers for *pimR*) and C (primers for *rrnA1*). Data are from three replicates and values represent the mean ± SD. Panel D summarizes information obtained from each plot.
(TIF)

Table S1 **Primers used in 5**′ **RACE experiments.**
(DOC)

Table S2 **Primers used in quantitative real-time PCR experiments.**
(DOC)
