# Supplementary figures and images for: Hierarchical Control on Polyene Macrolide Biosynthesis: PimR Modulates Pimaricin Production via the PAS-LuxR Transcriptional Activator PimM
Source: PLoS One. 2012 Jun 5;7(6):e38536. doi: 10.1371/journal.pone.0038536 (PMC3367932; doi:10.1371/journal.pone.0038536)

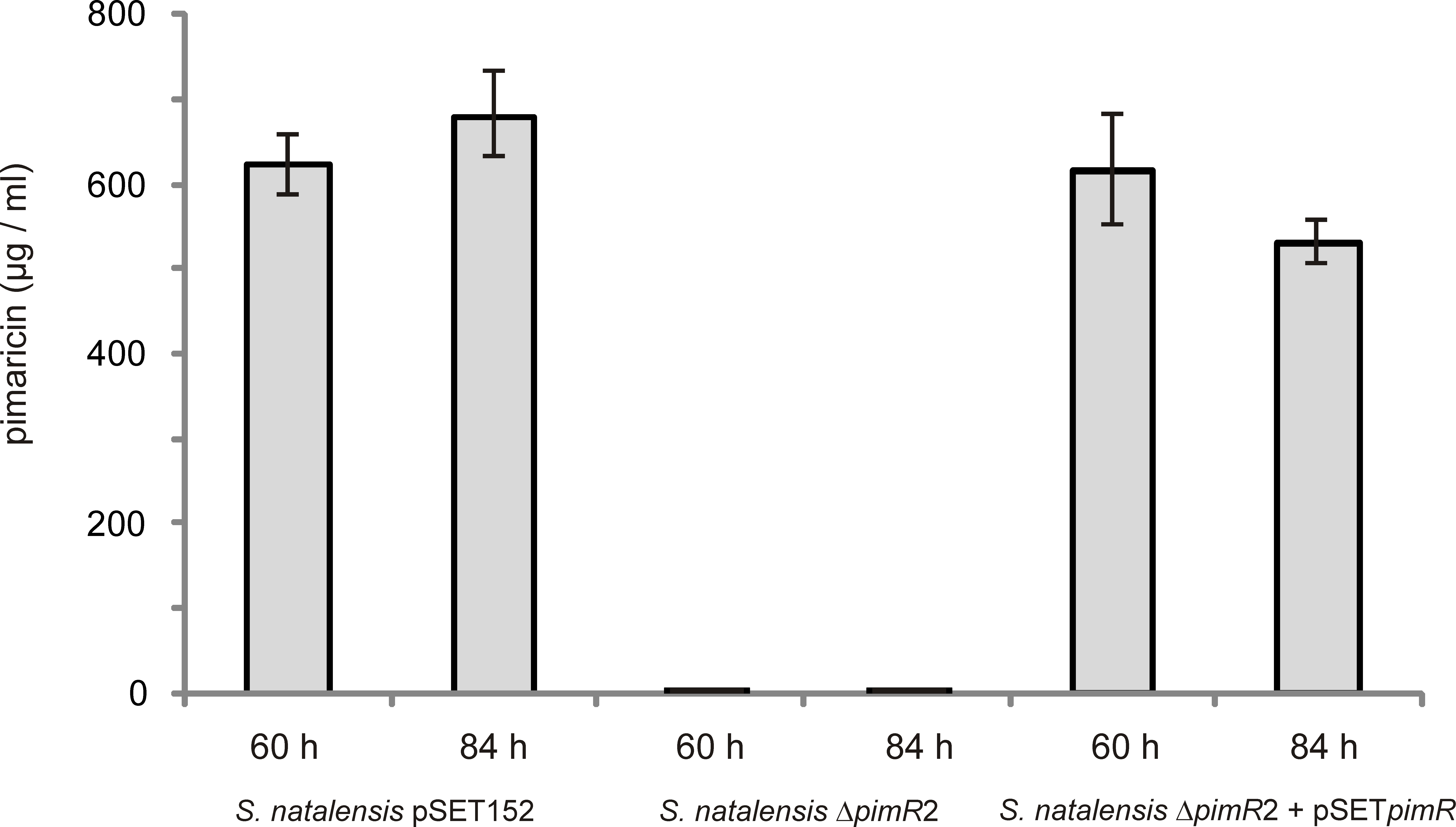

Supplement: Figure S1 — Gene complementation of S. natalensis ΔpimR2 mutant restores pimaricin biosynthesis. Quantification of the pimaricin production attained by the complemented strain after 60 and 84 hours of growth. Data are the average of three flasks. Vertical bars indicate the standard deviation values. (TIF) [file pone.0038536.s001.tif]

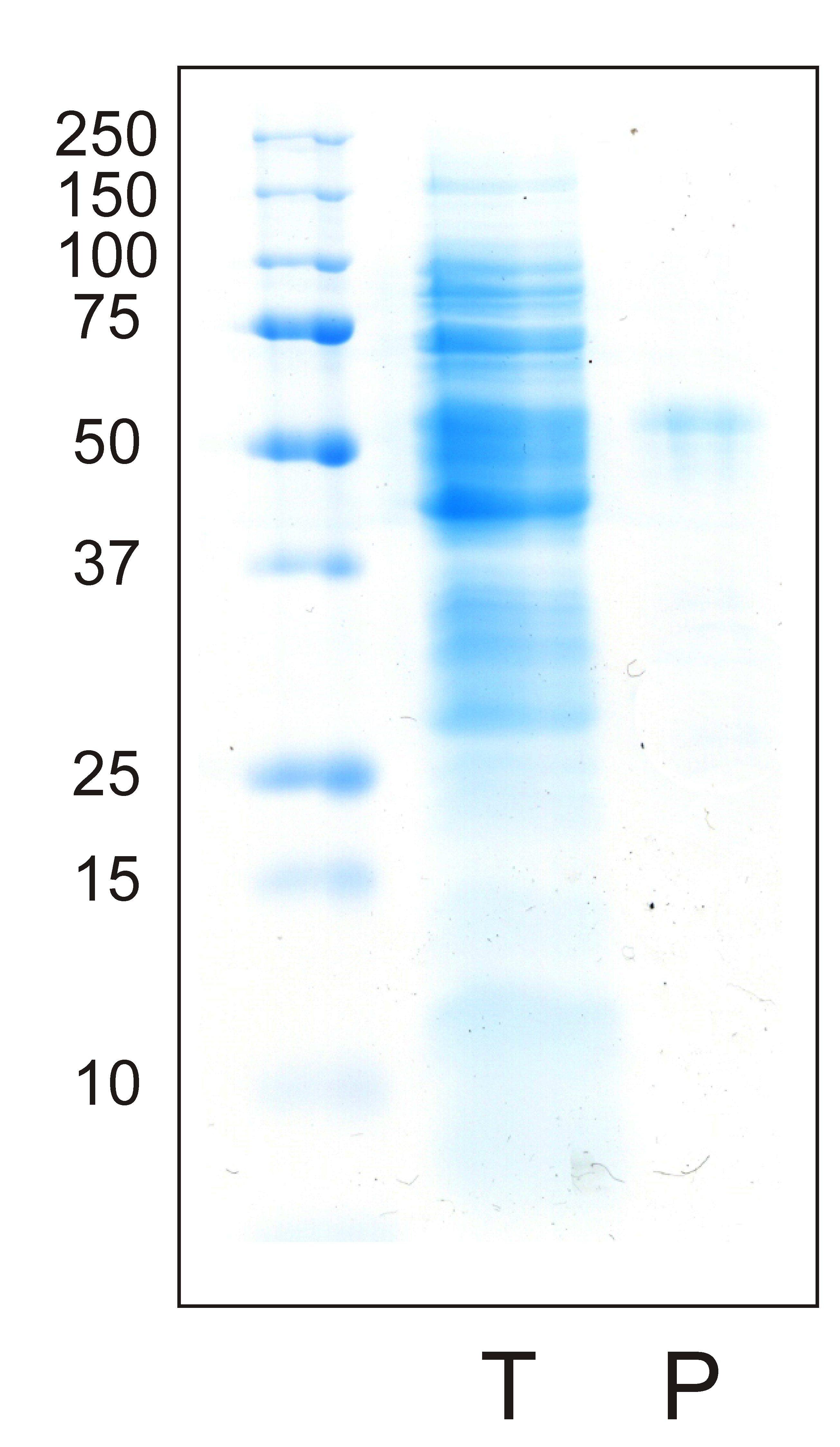

Supplement: Figure S2 — Purification of GST-fusion protein in E.coli BL21. Purification of GST-PimRSARP by affinity chromatography on Glutathione Sepharose. Lane T, total E. coli cell extract; lane P, purified proteins after affinity chromatography. Left lane, molecular size markers (in kDa). (TIF) [file pone.0038536.s002.tif]

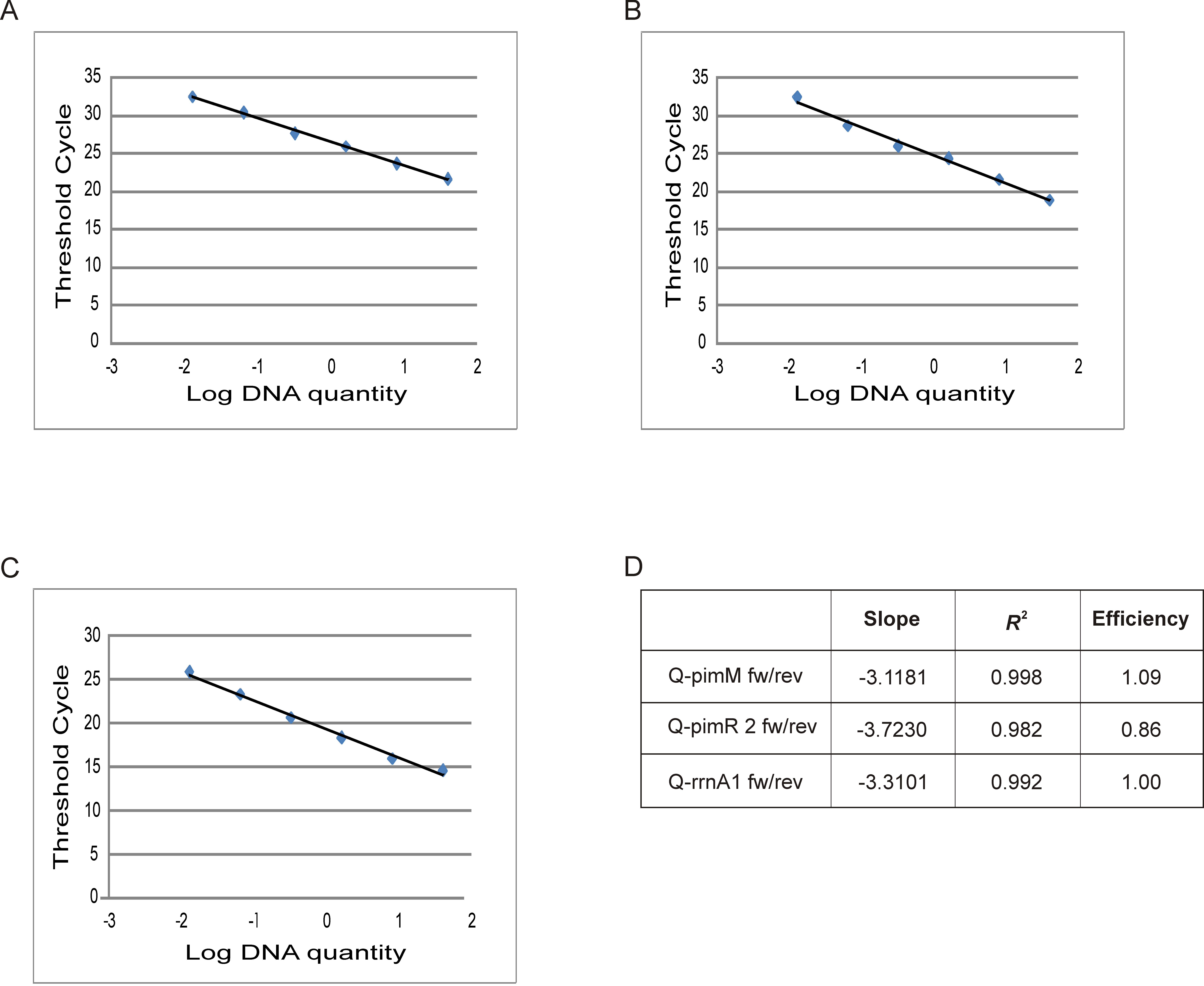

Supplement: Figure S3 — Primer efficiency. The efficiency of each set of primers was calculated according to the equation E = 10[−1/slope]−1. Using 5-fold dilutions of genomic DNA, the resulting Ct values were plotted against the logarithm of the DNA quantity as shown in A (primers for pimM), B (primers for pimR) and C (primers for rrnA1). Data are from three replicates and values represent the mean ± SD. Panel D summarizes information obtained from each plot. (TIF) [file pone.0038536.s003.tif]
