# Supplementary material for: Hierarchical Control on Polyene Macrolide Biosynthesis: PimR Modulates Pimaricin Production via the PAS-LuxR Transcriptional Activator PimM
Source: PLoS One. 2012 Jun 5;7(6):e38536. doi: 10.1371/journal.pone.0038536 (PMC3367932; doi:10.1371/journal.pone.0038536)
Supplement: Table S1 — Primers used in 5′ RACE experiments. (DOC) [file pone.0038536.s004.doc]

**Table S1: Primers used in 5´ RACE experiments**.

| **Primer** | **Sequence (5´ to 3´)** | **Transcription initiation site determination** |
| --- | --- | --- |
| M-1 | CAAGGGGACGGTGGAGACGCC |  |
| M-2 | GTGAGGGGAACGGTGAAGGCG | *pimM* |
| M-3 | CGGCGATGACGGGCGTGAC |  |
| R-1 | CCAGCAGCCGCCGTCCGTC |  |
| R-2 | GCGAGACATGGCTTTGGAGTGTGG | *pimR* |
| R-3 | CGTCACCCCACAGTTCCTCCACC |  |
