# Supplementary material for: Hierarchical Control on Polyene Macrolide Biosynthesis: PimR Modulates Pimaricin Production via the PAS-LuxR Transcriptional Activator PimM
Source: PLoS One. 2012 Jun 5;7(6):e38536. doi: 10.1371/journal.pone.0038536 (PMC3367932; doi:10.1371/journal.pone.0038536)
Supplement: Table S2 — Primers used in quantitative real-time PCR experiments. (DOC) [file pone.0038536.s005.doc]

**Table S2:** Primers used in quantitative real-time PCR experiments.

| Primer name | *Sequence* | *Description* | *Product size (bp)* |
| --- | --- | --- | --- |
| Q-rrnA1 fw | gacgcaacgcgaagaacc | Used to quantify *rrnA1* transcripts | 137 |
| Q-rrnA1 rev | tgcgggacttaacccaacatc |  |  |
| Q-pimR 2 fw | ccagccagctcgaatccg | Used to quantify *pimR* transcripts | 62 |
| Q-pimR 2 rev | cattacggtccccgtgc |  |  |
| Q-pimM fw | ttggagaattgacgcaccag | Used to quantify *pimM* transcripts | 125 |
| Q-pimM rev | atccagcaggcgaaccag |  |  |
